# Combination therapy of flaxseed and hesperidin enhances the effectiveness of lifestyle modification in cardiovascular risk control in prediabetes: a randomized controlled trial

**DOI:** 10.1186/s13098-020-00619-y

**Published:** 2021-01-06

**Authors:** Zahra Yari, Zahra Naser-Nakhaee, Elahe Karimi‐Shahrbabak, Makan Cheraghpour, Mehdi Hedayati, Seyede Marjan Mohaghegh, Shahrzad Ommi, Azita Hekmatdoost

**Affiliations:** 1 Department of Clinical Nutrition and Dietetics, Faculty of Nutrition and Food Technology, National Nutrition and Food Technology, Research Institute Shahid Beheshti University of Medical Sciences, Tehran, Iran; 2 Human Nutrition Department, McGill University, Montreal, QC Canada; 3 Cancer Research Center, Ahvaz Jundishapur University of Medical Sciences, Ahvaz, Iran; 4 Cellular and Molecular Endocrine Research Center, Research Institute for Endocrine Sciences, Shahid Beheshti University of Medical Sciences, Tehran, Iran; 5 Diabetes Research Center, Health Research Institute, Ahvaz Jundishapur University of Medical Sciences, Ahvaz, Iran; 6 Department of Dietetics and Nutrition, Florida International University, Miami, FL USA

**Keywords:** prediabetes, flaxseed, hesperidin, lifestyle modification, cardiometabolic

## Abstract

**Background:**

Regarding the increasing prevalence of cardiometabolic abnormalities, and its association with non-communicable chronic diseases, providing preventive and therapeutic strategies is a priority. A randomized placebo-controlled study was conducted to assess the effects of combination therapy of milled brown flaxseed and hesperidin during lifestyle intervention on controlling cardiovascular risk in prediabetes.

**Methods:**

A total of forty-eight subjects were randomly assigned to receive lifestyle intervention plus combination therapy of brown flaxseed (30 g milled) and hesperidin (two 500 mg capsules) or lifestyle modification alone for 12 weeks. Changes from baseline in anthropometric measures, lipid profile and atherogenic indices, glucose homeostasis parameters, and inflammatory biomarkers was assessed as a primary end point.

**Results:**

Anthropometric data comparison between the two groups showed a significant reduction in weight (*p *= 0.048). Waist circumference reduction was about twice that of the control group (− 6.75 cm vs − 3.57 cm), but this difference was not statistically significant. Comparison of blood pressure changes throughout the study indicated a greater reduction in blood pressure in the intervention group rather than control group (− 5.66 *vs.* − 1.56 mmHg, *P* = 0.049). Improvements of lipid profile and atherogenic indices, glucose homeostasis parameters, and inflammatory biomarkers in flaxseed-hesperidin group was significantly more than the control group after 12 weeks of intervention (*p *< 0.05).

**Conclusion:**

Our results indicate that co-administration of flaxseed and hesperidin as an adjunct to lifestyle modification program is more effective than lifestyle modification alone in the metabolic abnormalities remission of prediabetic patients.

*Trial registration: *The trial was registered with ClinicalTrials.gov, number NCT03737422. Registered 11 November 2018. Retrospectively registered, https://clinicaltrials.gov/ct2/results?cond=&term=NCT03737422&cntry=&state=&city=&dist=.

## Introduction

Type 2 diabetes mellitus (T2DM) and prediabetes (PD) are nowadays serious health problems all over the world and are usually accompanied with other metabolic disorders such as obesity, nonalcoholic fatty liver disease and cardiovascular diseases, mainly due to insulin resistance [1].

The prevalence of people with prediabetes in the world is reported to be more than 25%, all of whom are at risk for diabetes and its complications [2]. In addition to preclinical impaired glucose regulation, changes in insulin production, secretion from the pancreatic β-cells and its function in skeletal muscle, adipose tissue and liver are some of the prominent hallmarks of pre-diabetes and T2DM [3]. Lifestyle modifications (LSM) including increasing exercise and optimizing dietary composition have been proposed as a therapeutic and preventive strategy for PD [4]. However, it has been shown that even though lifestyle modification strategies are adapted, many people with pre-diabetes will progress to T2DM [5].

Flaxseed and hesperidin may improve cardiovascular health due to their numerous attributes [6, 7]. Hesperidin and flaxseed have emerged as promising phyto-therapeutic agents, which may exert potential benefits on health mainly through improving the antioxidant status [8], and providing adequate fiber and omega-3 [9–11], respectively. The therapeutic effects of these two compounds on metabolic dysregulation in PD can be attributed to their powerful biological properties on improving glucose homeostasis and insulin resistance [12–15].

Recently, several studies have shown anti-diabetic [15, 16] and anti-inflammatory [17] properties of hesperidin and flaxseed. Intake of omega-3 fatty acids and fiber, both of which are compounds of flaxseed, can contribute to reducing the risk of metabolic abnormalities [9, 18, 19]. Beneficial effects of flaxseed on lowering plasma glucose in patients with diabetes as well as metabolic syndrome have been reported [20–22]. Similar effects have been obtained with the consumption of flax lignan [21]. Although hesperidin exerts many health benefits [23], its effects on blood glucose have been inconsistent in various studies, as in four studies [24–27], no effect was detected and in one study [28], blood sugar was reduced.

Although the beneficial effects of flaxseed and hesperidin on cardiometabolic risk factors have been shown previously, no study has evaluated the effects of their combinations on pre-diabetic patients. Considering lack of data on such combination therapies in patients with PD, we aimed to investigate whether patients following LSM program along with supplementing with 30 g milled brown flaxseed and one g hesperidin per day, would have better metabolic parameters compared with those who just follow the LSM program.

## Methods and materials

### Subjects

To assess the effects of combination therapy of milled brown flaxseed and hesperidin during lifestyle modification on cardiovascular risk in PD patients, 80 men and women with PD were recruited from a nutrition clinic by the principal investigators and by using advertisements. For study inclusion, participants were required to be aged 18 to 70 years and diagnosed with PD based on impaired fasting glucose (≥ 100 mg/dL), impaired glucose tolerance (2-hour glucose level 140 to 200 mg/dL after ingesting 75 g of oral glucose) and/or HbA1C 5.7% to 6.5% [29] and having body mass index (BMI) range of 25 to 40 kg/m2. The exclusion criteria were any history of cardiovascular, pulmonary, renal, hepatic and gastrointestinal disease. Additionally, taking anti-hypertensive, glucose-lowering medications, lipid modifying agents and omega-3 dietary supplement, being under treatment with steroids and nonsteroidal anti-inflammatory drugs, being on calorie-restricted regimen within 3 months before the commencement of the trial, being pregnant, lactating were the other main exclusion criteria.

**Fig. 1:**
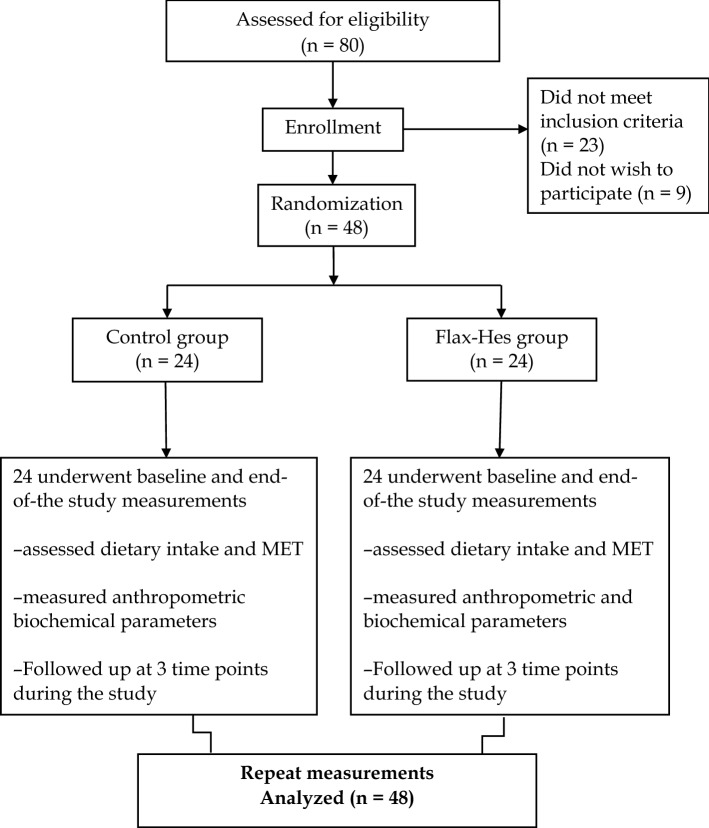
Flow chart depicting the study design

Of the 80 subjects screened, 57 patients including 31 females and 26 males were deemed eligible for inclusion; of which nine were reluctant to participate in the trial. Eventually, 48 patients were enrolled. A flow diagram of the trial is presented in Fig. 1. Twenty-four patients consumed 30 g/day milled brown flaxseed two 500 mg capsules of hesperidin along with LSM and 24 patients followed LSM program for 12 weeks.

### Study design

We conducted a randomized, open labeled, controlled study on patients with prediabetes. The trial was registered with ClinicalTrials.gov, number NCT03737422.

Ethical approval was granted by the Ethical Committee of Shahid Beheshti University of Medical Science (IR.SBMU.RETECH.REC.1398.561), and all procedures were in accordance with the Helsinki Declaration. Participation was voluntary, and all patients provided written informed consent, before beginning the study.

The participants were recruited from advertisement and primary health care centers between January 2018 and July 2018. One of the research assistants who had no other involvement in the trial randomly allocated patients to groups. Since the study was designed as open-labeled, neither patients nor researchers were blind to the interventions. All patients underwent follow-up visits at 4, 8, and 12 weeks after enrolment, at which dietary advice was repeated to patients and adequate supplements were given. Participants were explicitly counseled regarding supplement consumption and how to follow the LSM program, before initiation of the trial.

### Supplementation

Each patient was assigned to one of two treatment groups using a table of random numbers for twelve-week intervention of either: (1) lifestyle modification program, (2) lifestyle modification plus combination therapy of brown flaxseed (30 g milled) and hesperidin (two 500 mg capsules). Also, patients were instructed to divide supplements into two equal doses (one capsule of hesperidin and 15 g milled flaxseed), one with breakfast and the other at lunchtime.

Hesperidin was 95% pure. The chemical compounds content of flaxseed is well-known. An analysis of brown-seeded flaxseed showed about 41% fat, 20% protein, 28% total dietary fiber and 3.4% mineral-rich ash [11].

### Blood sampling and laboratory tests

Blood samples (7 mL) were collected in the morning after overnight fasting, before and after the intervention, for biochemical measurements. After centrifugation of blood at 3700 rpm for 10 min at room temperature, the separated serum was stored frozen at − 80 °C until the tests were run.

The levels of serum HDL-C (high density lipoprotein cholesterol) and TG (triglyceride) were measured by the standard enzymatic method (Pars Azmoon kit, Tehran, Iran). Fasting serum glucose was analyzed by the glucose-oxidase method (Pars Azmoon Co., Tehran, Iran) and enzyme-linked immunosorbent assay (ELISA) was applied for measuring fasting insulin (Monobind Inc., USA), high sensitive C-reactive protein (hs-CRP) (Zellbio, Germany), and tumor necrosis factor alpha (TNF-α) (Diaclone Inc., France). Low density cholesterol (LDL-C) level was determined using the Friedewald Eq.  [30]. Insulin resistance was calculated by using this formula: Fasting Glucose (mg/dl) x fasting Insulin (µU/mL)/405 [31]. Also, the following formula was used to calculate insulin sensitivity:1/[log glucose (mg/dL) + log insulin (µU/mL)] [32].

In addition to lipid profile assessing, some indicators including non-HDL/HDL ratio, LDL/HDL ratio and atherogenic index of plasma (AIP) were calculated to investigate the atherogenicity. AIP, as a predictor of the risk of cardiovascular disease, is calculated by logarithmically transformation of triglycerides to HDL cholesterol ratio. Accordingly, it is inversely correlated with the size of LDL particles [33].

### Anthropometrics measurements

A clinical evaluation including medical history and clinical data sheet was performed. All participants underwent a detailed anthropometric assessment at the baseline and after completion of the intervention.

Height and weight were measured by a trained research assistant based on the standard protocol using calibrated Seca scale with attached stadiometer and recorded to the nearest 0.5 cm and 0.1 kg, respectively. For body mass index calculation, weight in kilograms divided by the square of height in meters. Minimum waist circumference was measured with a tape to the nearest 0.5 cm so that we could properly identify subjects who had central obesity.

### Dietary assessment and physical activity

For dietary assessment, we completed three 24-hour food recall questionnaires at the baseline and after the 12-week study intervention for each patient. Dietary intakes were then analyzed using the nutritionist version 4 (N4) software.

To assess physical activity level, a questionnaire was completed for each patient, which included a list of daily activities, the frequency and time of activities spent per day. Physical activity levels were expressed as metabolic equivalent (METs h/day).

### Blood pressure

After 15 min of resting, blood pressure was measured twice on the right arm, using a standard mercury sphygmomanometer [34], at baseline and at 12 weeks with a standard sphygmomanometer. The average of two measurements with at least 10-min interval was recorded.

### Data analysis

All analyses were performed using the statistical package for social sciences software (IBM SPSS Statistics for Windows, release 21.0. Armonk, NY, USA: IBM Group). Before further analysis, normal distribution of the variables was checked by applying the Shapiro–Wilk test. Comparison of changes before and after intervention in each group were analyzed by paired *t* test. In addition, as appropriate, student’s *t* test and analysis of covariance (ANCOVA) were applied to assess differences between two groups using baseline value of the outcome, age, sex and mean changes in BMI, MET and energy as covariates. P < 0.05 was considered as the significance level.

## Results

A total of 80 patients completed the initial screening, of which 32 were excluded due to a lack of interest and failure to meet the entrance criteria. Finally, 48 patients received the allocated intervention. All patients completed a 12-week follow-up program, with an overall intervention adherence rate of 100%. Flow diagram of the study is presented in Fig. 1.

As shown in Table 1, there was no significant difference between two groups with regard to baseline characteristics including anthropometric measurements, blood pressure and serum biochemistry tests. There were 25 men (52%), and 23 women (48%) among all participants. The mean age of participants was 44.5 ± 10.7 and 46.7 ± 11.5 in intervention and control group, respectively. No significant differences among the distributions of sex and age between two groups were observed.

**Table 1:** Baseline characteristics at enrollment

Characteristics	Total (n = 48)	Flax-Hes group (n = 24)	Control group (n = 24)	*P* value
Age (y)	45.63 ± 11.00	44.50 ± 10.66	46.75 ± 11.46	0.485
Sex (M/F)	25/23	12/12	13/11	1.000
Metabolic characteristics				
Height (cm)	166.96 ± 10.54	167.38 ± 9.03	166.54 ± 12.04	0.787
Weight (kg)	91.24 ± 14.96	92.08 ± 16.06	90.40 ± 14.08	0.700
WC (cm)	104.71 ± 7.50	105.25 ± 6.43	104.17 ± 8.55	0.622
BMI (kg/m2)	33.09 ± 5.42	33.27 ± 5.43	32.92 ± 5.51	0.826
Blood pressure (mmHg)				
Systolic	128.73 ± 14.94	129.71 ± 14.77	127.80 ± 15.35	0.660
Diastolic	85.24 ± 12.93	86.21 ± 14.66	84.32 ± 11.26	0.615
MET (h/d)	31.93 ± 4.45	32.65 ± 2.97	31.22 ± 5.54	0.270
Energy (kcal)	2473.56 ± 487.94	2535.15 ± 540.39	2411.98 ± 431.96	0.388
Serum biochemistry tests				
FBS (mg/dL)	113.05 ± 19.61	117.08 ± 21.16	107.96 ± 16.62	0.123
Insulin (mU/L)	13.18 ± 7.17	12.58 ± 6.85	13.94 ± 7.67	0.545
HOMA-IR	3.69 ± 2.34	3.78 ± 2.57	3.57 ± 2.10	0.773
QUICKI	0.33 ± 0.03	0.33 ± 0.03	0.32 ± 0.02	0.902
Triglyceride (mg/dL)	162.02 ± 71.27	159.83 ± 79.09	164.78 ± 61.98	0.824
HDL-C (mg/dL)	35.51 ± 9.69	36.15 ± 9.82	34.68 ± 9.73	0.629
LDL-C (mg/dL)	138.36 ± 37.53	140.18 ± 42.21	135.93 ± 31.25	0.721
hs-CRP (ng/dL)	5865.44 ± 4564.82	6879.25 ± 5609.71	4584.84 ± 3447.53	0.102
TNF-α (pg/mL)	23.60 ± 7.63	25.10 ± 8.84	21.49 ± 4.98	0.138
Atherogenic indices				
Atherogenic index of plasma (AIP)	0.64 ± 0.25	0.61 ± 0.27	0.67 ± 0.24	0.515
Non-HDL/HDL ratio	5.28 ± 2.19	5.25 ± 1.81	5.33 ± 2.67	0.910
LDL/HDL ratio	4.25 ± 1.78	4.15 ± 1.62	4.41 ± 1.99	0.645

*WC* waist circumference, *BMI* body mass index, *MET* metabolic equivalent of tasks, *FBS* fasting blood sugar, *HOMA*-*IR* The homeostatic model assessment, *QUICKI* quantitative insulin sensitivity check index, *HDL*-*C* high density lipoprotein, *LDL*-*C* low density lipoprotein

Data are shown as Mean ± SD

Furthermore, data on diet indicated no significant differences in total energy, macronutrients or micronutrients intake between groups. Although the results of the paired t-test revealed a significant decrease in energy intake and macronutrients, except for protein, throughout the study in both groups; but this difference between the two groups was not significant.

A paired t-test was used to compare the changes in each group during the 12-week intervention period. All of the measurements in flax-hes group were significantly improved, except for HDL-C and hs-CRP. In control group, in addition to these two factors, systolic blood pressure and serum triglyceride, AIP and non-HDL/HDL ratio did not show significant changes (Table 2).

**Table 2:** Mean changes (95% CI) from baseline in metabolic characteristics by treatment group

Change from baseline	Flax-Hes group (n = 24)	Control group (n = 24)	*P* value^a^
Weight (kg)	− 5.10 (− 6.45, − 3.76)	− 3.10 (− 4.65, − 1.55)	0.048
*P* value^b^	< 0.001	0.001	
WC (cm)	− 6.75 (− 10.53, − 2.97)	− 3.57 (− 6.43, − 0.73)	0.306
*P* value^b^	0.001	0.017	
Systolic blood pressure	− 5.66 (− 10.53, − 1.29)	− 1.56 (− 3.57, 0.45)	0.049
*P* value^b^	0.013	0.123	
Diastolic blood pressure	− 3.83 (− 8.43, 0.76)	− 2.92 (− 5.65, − 0.18)	0.456
*P* value^b^	0.048	0.037	
FBS	− 20.35 (− 28.07, − 12.63)	− 7.46 (− 12.55, − 2.37)	0.007
*P* value^b^	< 0.001	0.007	
Insulin	− 3.43 (− 4.86, − 1.99)	− 2.46(− 4.21, − 0.72)	0.373
*P* value^b^	<0.001	0.008	
HOMA-IR	− 1.59 (− 2.37, − 0.83)	− 0.73 (− 1.22, − 0.24)	0.055
*P* value^b^	< 0.001	0.006	
QUICKI	0.03 (0.01, 0.04)	0.01 (0.004, 0.014)	0.023
*P* value^b^	0.001	0.001	
Triglyceride	− 45.30 (− 64.16, − 26.43)	− 8.39 (− 43.40, 26.62)	0.020
*P* value^b^	< 0.001	0.620	
HDL-C	2.73 (− 1.13, 6.59)	1.30 (− 1.36, 3.96)	0.535
*P* value^b^	0.155	0.317	
LDL-C	− 38.35 (− 55.72, − 20.97)	− 12.73 (− 24.11, − 1.36)	0.016
*P* value^b^	< 0.001	0.030	
hs-CRP	− 1044.22 (− 2169.84, 81.39)	− 696.05 (− 2642.58, 1250.46)	0.350
*P* value^b^	0.067	0.459	
TNF-α	− 6.52 (− 9.83, − 3.21)	− 1.95 (− 3.61, − 0.29)	0.015
*P* value^b^	0.001	0.024	
Atherogenic index of plasma (AIP)	− 0.16 (− 0.21, − 0.1)	− 0.03 (− 0.15, − 0.09)	0.039
*P* value^b^	< 0.001	0.539	
Non-HDL/HDL ratio	− 1.17 (− 1.67, − 0.66)	− 0.59 (− 1.38, 0.18)	0.042
*P* value^b^	< 0.001	0.121	
LDL/HDL ratio	− 1.16 (− 1.74, − 0.58)	− 0.63 (− 1.14, − 0.11)	0.076
*P* value^b^	< 0.001	0.020	

^a^Based on an ANCOVA model that regressed changes from baseline on treatment group, baseline value of the outcome, age, sex and mean changes in BMI, MET and energy

^b^Paired t-test

The difference of changes between two groups during the interventions were analyzed using ANCOVA adjusted for baseline value of the outcome, age, sex and mean changes in BMI, MET and energy intake.

Mean changes of metabolic characteristics of participants from baseline are presented in Table 2. Anthropometric data comparison between the two groups showed a significantly more reduction in weight in intervention group compared to placebo group (*P *= 0.048). With regard to waist circumference, although in the intervention group, WC reduction was about twice that of the control group (− 6.75 cm vs − 3.57 cm), but this difference was not statistically significant (*P *= 0.306).

Comparison of blood pressure changes throughout the study between the two groups indicated a greater reduction in blood pressure in the intervention group, although this difference was significant only for systolic blood pressure (*P* = 0.049). Regarding the glucose homeostasis factors, only significant differences were observed in the values of FBS and QUICKI (*P *= 0.007, and *P *= 0.023, respectively). Also, we found approximately near to the significant level differences in HOM-IR (*P* = 0.055). However, the changes in insulin levels were fairly similar in both groups (Table 2).

Based on the results presented in Table 2, the reduction in TG and LDL was significantly more in flax-hes group than the control group after 12 weeks of intervention (*P *= 0.020, and *P *= 0.016, respectively).. Although HDL levels increased within both groups, there was no significant difference between the two groups. Improving atherogenicity indices in the intervention group was significantly superior to the control group (*P *= 0.039). Coadministration of flaxseed and hesperidin could significantly reduce AIP, LDL/HDL ratio and non-HDL/HDL ratio over a 12-week period. While in the control group, only LDL/HDL ratio reduction was significant.

Also, a significant decrease in TNF-α indicates that supplementation with hesperidin and flaxseed in comparison with LSM alone (− 6.5 vs − 1.9 pg/mL) was observed (*P *= 0.015).

### Adverse events and compliance

Patients were instructed to return any unused supplements at every follow up visit. None of the patients had missed more than 10% of the supplements. Also, no complication or serious side effects were observed during the study among the participants that could be attributed to intervention.

## Discussion

We found that including daily intake of 30 g of whole flaxseed and one gram of hesperidin can modulate cardiometabolic risk factors in patients with prediabetes. The combination of hesperidin, as a flavonoid, and flaxseed, as a functional food, can exhibit a great potency in preventing and controlling cardiometabolic abnormalities through their combined anti-inflammatory and anti-oxidant properties as well as high fiber content. Reducing the risk of cardiometabolic complications has been stem from increasing insulin sensitivity, improving dyslipidemia and lowering blood pressure succeeding consumption of flaxseed and hesperidin.

In the present study, although the concentration of HDL did not change, a decline in the concentration of triglyceride and LDL was observed in response to hes-flax supplementation. These findings are consistent with preceding studies. Administration of 500 mg of purified hesperidin to patients with hypertriglyceridemia markedly reduced serum triglyceride levels over a 24-week period [35], while 800 mg of hesperidin failed to affect plasma HDL-cholesterol after 4 weeks [36]. Though in another study, oral administration of 500 mg hesperidin for 3 weeks resulted in a significant increase in HDL, while failed to reduce triglyceride levels [37]. One of the proposed mechanisms of the hesperidin effects on lipid profile modulation is through increasing the transcription of the peroxisome proliferator-activated receptor alpha (PPAR-α) gene [38]. In order to achieve significant clinical outcomes, flavonoids should be sufficiently consumed. Moreover, the patient population and length of study are other imperative determinants. In fact, lipid-lowering effects of purified hesperidin should be interpreted with caution as this may not be achievable with usual dietary intake [38].

On the other hand, flaxseed has beneficial effects on lipid profile and atherogenicity, which are due to its components specially fiber and omega-3 fatty acids. A recent clinical trial revealed that adding 10 g flaxseed pre-mixed in cookies twice a day to the usual diet significantly ameliorated the serum levels of cholesterol, LDL-C and cholesterol/HDL ratio in patients with T2DM. Their results indicate that the benefits of flaxseed in weight reduction, lipid profile control and glucose homeostasis is superior to those of psyllium [39]. Also, it was reported in a study conducted in diabetic patients that daily consumption of flaxseed enriched yogurt (containing 30-g flaxseed), although could not alter serum LDL-C and HDL-C, decreased serum triglycerides and total cholesterol significantly [40]. Moreover, high content of flaxseed lignan can reduce triglyceride levels through suppressing the sterol regulatory element-binding proteins (SREBP) mRNA [41]. It has been revealed that the hypocholesterolemic effect of flaxseed is more pronounced than wheat in hypercholesterolemic adults, though it led to a slight decrease in HDL-C concentration [42]. What seems to have arisen from this study is that fiber alone cannot fully account for all the positive effects of flaxseed in improving the lipid profile. At least part of these beneficial effects is due to omega-3 fatty acids, which has been mentioned in previous studies [43, 44]. However, the findings of some studies are inconsistent with these results [45], possibly because the dose or duration of the intervention was insufficient. Since it has been reported that at least consumption of 30 grams flaxseed is required to convert alpha linolenic acid (ALA) to eicosapantanoic acid (EPA) in young, healthy adults [46]. Thus, it seems that the synergistic effects of flaxssed and hesperidin reduced TG and LDL in our study.

One of the most remarkable findings of the present study was the improvement of glycemic parameters, including fasting plasma glucose, insulin, HOMA-IR and QUICKI after 12 weeks treatment with combination of hesperidin and flaxseed, which is in line with the findings of most previous studies [24, 47, 48]. Although no study has evaluated the effects of combination of these supplements, there are several studies that have shown the beneficial effects of each of them alone on glycemic homeostasis.

A study by Cunnane et al. reported the hypoglycemic effects of both whole flaxseed and isolated flaxseed fiber [22]. Improvements in either HOMA-IR or QUICKI following flaxseed consumption was described for the first time in 2013 [42]. The prominent point of these studies is that flaxseed supplementation can only affect glycemic parameters in interventions duration of at least 12 weeks. The study conducted by Barre and colleagues [21] is in line with this finding, in which 3 months supplementation of flaxseed lignan in patients with type 2 diabetes led to plasma glucose reduction. This was also confirmed in another study in which flaxseed consumption in patients with metabolic syndrome could result in blood glucose control and insulin resistance decline after 12 weeks [20]. Also, a recently published meta-analysis in this area also indicated this, but with this difference that only whole flaxseed, but not flaxseed oil and lignan extract, has significant effects on controlling glycemic parameters [49]. A possible explanation for the necessity of long-term intervention (≥ 12 weeks) is that improving glucose control and insulin sensitivity require an increase in EPA and Docosahexaenoic acid (DHA) concentrations [50, 51] and ALA conversion to these two fatty acids is time-consuming [52]. Also, the function of the gut microflora is enhanced gradually by flaxseed fiber consumption, which in turn led to improved blood glucose control and insulin function [53, 54]. As well, the higher initial concentration of plasma glucose (> 100 mg/dL), the more pronounced changes can be achieved via the flaxseed supplement [49].

On the other hand, according to the Nurses’ Health Studies (NHS) I and II, higher urinary excretion of hesperetin is associated with a lower middle- and long-term risk of type 2 diabetes (OR: 0.68) [55]. Improving insulin sensitivity and reducing insulin resistance has been stated repeatedly after treatment with hesperidin [24, 37, 56]. Increased skeletal muscle glucose uptake has been shown following the positive regulation of insulin signaling by hesperidin. That is why intake of orange juice containing hesperidin following a high-fat and high-carbohydrate meal, prevents increases in the expression of suppressor of cytokine signaling-3, a negative modulator of insulin signaling [57]. Thus, combination of hesperidin and flaxseed had synergistic effects on reduction of blood glucose in our study.

Our results demonstrated that patients supplementing with the combination of flaxseed and hesperidin experienced lowered systolic blood pressure after 12 weeks. The anti-hypertensive effect of flaxseed and hesperidin has been shown in previous studies. In the same way, a study conducted in 2015 found that higher urinary polyphenol excretion was allied with lower systolic and diastolic blood pressure, which was attributed to nitric oxide production [58, 59]. A meta-analysis of the effects of flaxseed on blood pressure showed that supplementation of various products of the flaxseed can exert beneficial effects on systolic and diastolic blood pressure control [60]. This study stated that flaxseed can cause a decrease of 2.85/2.39 mmHg in blood pressure. Since patients with prediabetes are at high risk for cardiovascular disease, this finding is very valuable. Similarly, Heart Outcome Evaluation study proposed that a 3.3/1.4 mmHg reduction in blood pressure is associated with a 22% decline of relative risk of cardiovascular mortality [61].

It is supposed that flaxseed lignan can possess an inhibitory action on angiotensin-converting enzyme (ACE) which results in blood pressure reduction [62]. Also, ALA in flaxseed suppresses the soluble epoxide hydrolase activity, which reduces blood pressure through inhibiting the production of inflammatory oxylipins and following vasoconstriction, inflammation and hypertension [63]. Although the blood pressure lowering effects of flaxseed have been attributed to fiber and ALA, the exact mechanisms involved in this effect are still not fully understood. Although bioavailability of ALA in whole flaxseed is lower than that of flaxseed oil, whole grain flaxseed has the advantage of providing all bioactive components and fiber with well tolerability [64, 65]. Although in our study, combination of flaxseed and hesperidin reduced more than previous studies that used them individually [66, 67], we did not find a significant difference between two groups in DBP. This might be due to receiving the lifestyle modification in control group, which caused a significant decrease in DBP of them.

Undeniably, obesity and overweight as well as central obesity are important risk factors for cardiometabolic diseases [68]. Our study showed significant reduction in weight and waist circumference after 12 weeks supplementation with the combination of flaxseed and hesperidin, although the difference of WC was not significant between two groups, compared to baseline. Studies regarding the effect of hesperidin on anthropometric measures are limited. Supplementation with citrus juice containing vitamin C and hesperidin have no significant effect on weight and WC [56]. Therefore, it appears that the weight loss observed in present study is possibly due to the flaxseed, which is in line with previous studies [42, 69]. Recent review and meta-analysis study also confirms the positive effects of flaxseed on weight loss, especially if the intervention durations of ≥ 12 weeks [70].

ALA can exert its anti-obesity effects via subsequent converting into long chain poly unsaturated fatty acids in the body [71–73]. Furthermore, flaxseed lignans under the gut microflora produced enterolactone, the main component of mammalian lignans, which has been negatively correlated with obesity [74, 75].

The present study has several strengths; to the best of our knowledge, it is the first interventional study investigating the effect of flaxseed and hesperidin combination as an adjunct to lifestyle modification for the cardiovascular control in prediabetes. Although the exact clinical implication of these findings is yet undefined, it could be proposed as a preventive strategy. It needs further studies to explore full potential of flaxseed and hesperidin and confirm these findings.

## Conclusions

In conclusion, incorporating 30 g of brown flaxseed and one-gram hesperidin into a healthy diet along with increasing physical activity seems reasonable and valuable to reduce risk of cardiometabolic abnormalities through ameliorating insulin resistance, lowering blood pressure, improving glycemic control, modifying lipid profile, and reduction of inflammation [11, 76, 77].

## Data Availability

All data generated or analyzed during this study are included in this published article.

## Abbreviations

T2DM: Type 2 diabetes mellitus
PD: Prediabetes
LSM: Lifestyle modifications
BMI: Body mass index
HDL-C: High density lipoprotein cholesterol
TG: Triglyceride
ELISA: Enzyme-linked immunosorbent assay
hs-CRP: High sensitive C-reactive protein
TNF-α: Tumor necrosis factor alpha
LDL-C: Low density cholesterol
AIP: Atherogenic index of plasma
METs: Metabolic equivalent
ANCOVA: Analysis of covariance
WC: Waist circumference
FBS: Fasting blood sugar
HOMA-IR: The homeostatic model assessment
QUICKI: Quantitative insulin sensitivity check index
PPAR-α: Peroxisome proliferator-activated receptor alpha
SREBP: Sterol regulatory element-binding proteins
ALA: Alpha linolenic acid
EPA: Eicosapantanoic acid
DHA: Docosahexaenoic acid
NHS: Nurses’ Health Studies
ACE: Angiotensin-converting enzyme
